# Causal illusions in the classroom: how the distribution of student outcomes can promote false instructional beliefs

**DOI:** 10.1186/s41235-020-00237-2

**Published:** 2020-08-03

**Authors:** Kit S. Double, Julie Y. L. Chow, Evan J. Livesey, Therese N. Hopfenbeck

**Affiliations:** 1 Department of Education, University of Oxford, 15 Norham Gardens, Oxford, OX2 6PY UK; 2 University of Sydney, Sydney, NSW Australia

**Keywords:** Teacher beliefs, Outcome density effect, False beliefs, Causal illusions, Contingency learning

## Abstract

Teachers sometimes believe in the efficacy of instructional practices that have little empirical support. These beliefs have proven difficult to efface despite strong challenges to their evidentiary basis. Teachers typically develop causal beliefs about the efficacy of instructional practices by inferring their effect on students’ academic performance. Here, we evaluate whether causal inferences about instructional practices are susceptible to an *outcome density effect* using a contingency learning task*.* In a series of six experiments, participants were ostensibly presented with students’ assessment outcomes, some of whom had supposedly received teaching via a novel technique and some of whom supposedly received ordinary instruction. The distributions of the assessment outcomes was manipulated to either have frequent positive outcomes (high outcome density condition) or infrequent positive outcomes (low outcome density condition). For both continuous and categorical assessment outcomes, participants in the high outcome density condition rated the novel instructional technique as effective, despite the fact that it either had no effect or had a *negative* effect on outcomes, while the participants in the low outcome density condition did not. These results suggest that when base rates of performance are high, participants may be particularly susceptible to drawing inaccurate inferences about the efficacy of instructional practices.

## Significance statement

The article outlines a series of six experimental studies that examine whether biases in contingency learning affect the judgements participants make about the efficacy of a teaching technique. The manuscript shows an outcome density effect in judgements of novel teaching methods’ efficacy, whereby participants who are exposed to frequent positive student outcomes in a contingency learning task erroneously conclude that the teaching technique is effective. The study has implications for understanding why inaccurate beliefs are prevalent among educators and why such beliefs do not necessarily self-correct over time.

A number of widespread beliefs about instructional practice have been criticised as lacking a scientific basis (e.g. Dekker, Lee, Howard-Jones, & Jolles, 2012; Howard-Jones, 2014; Kirschner, 2017; Kirschner, Sweller, & Clark, 2006). For example, 93% of teachers still subscribe to the (now widely debunked) idea of student learning styles (Dekker et al., 2012). Like urban myths, many of these beliefs have persisted for a long time, often decades after a scientific consensus around their inaccuracy was reached (Kirschner, 2017). Inaccurate instructional beliefs are often adopted to the exclusion of more evidence-based practices, which has a significant negative effect on academic outcomes (Bruyckere, Kirschner, & Hulshof, 2015). While, such practices are often spread culturally, being passed from teacher to teacher, they are also often reinforced by inaccurate media stories, social media, and a lack of scientific education (Pasquinelli, 2012). Teacher misconceptions are remarkably robust across countries and pseudoscientific practices are increasing in schools worldwide (Ferrero, Garaizar, & Vadillo, 2016; OECD, 2002). Little is known, however, about the psychological mechanisms that reinforce inaccurate beliefs about instructional practice. Previous studies into contingency learning—how individuals learn about the statistical relationship between a behaviour and an outcome—have suggested that inaccurate beliefs about the causal effect of a behaviour can often form when the expected outcome occurs frequently, irrespective of the actual contingency between behaviour and the outcome – a so-called *outcome density effect* (Blanco, Matute, & Vadillo, 2013; Chow, Colagiuri, & Livesey, 2019). In the current study, we examine the outcome density effect in the context of teacher beliefs, by evaluating whether the distribution of students’ outcomes on an academic assessment (e.g. a class test) influences participants’ false beliefs about the efficacy of instructional practices.

## Contingency learning

Like most beliefs that people hold, teachers’ beliefs are often causal in nature, that is, they are motivated by perceived cause and effect relationships (e.g. If I use teaching practice *x* my students’ performance will improve). Teachers acquire these beliefs by accumulating evidence about the cause-effect relationship through direct experience with the putative cause (often referred to as the cue); e.g. the use of a novel teaching practice, and the desired outcome, e.g. improvement in grades. If the cue is influential in changing the outcome, then the probability of the outcome occurring should differ as a function of whether the cue was present or absent. The process of extracting causal information in this way is often referred to as *contingency learning* (Jenkins & Ward, 1965). This difference in the probability of events is more formally captured in Allan’s delta *p* (*Δp*) index (Allan, 1980):

1 $$ \Delta p=p\left(O|C\right)-p\left(O|\sim C\right) $$

Δp = contingency

p(O|C) = probability of the outcome given the cue

p(O|~C) = probability of the outcome given no cue

According to Eq. 1, a positive *Δp* indicates a positive contingency between cue and outcome, such that the probability of the outcome occurring is greater when the cue is present than when it is absent (i.e. the novel teaching practice is effective at improving students’ grades). In contrast, a negative *Δp* value indicates that the novel teaching practice is producing worse outcomes than if students were not given the novel teaching practice at all. The ability to extract causal information through experience is a necessary tool for navigating the world; people are motivated to produce behaviours that lead to a desirable outcome and avoid behaviours that produce undesirable ones. In fact, people are generally good at identifying positive and negative contingencies between events (e.g. Shanks & Dickinson, 1988); however, when there is no genuine relationship between the two events, that is *Δp* = 0, people tend to overestimate the causal relationship and develop a false causal belief. This phenomenon is often referred to as the illusion of causality or illusory causation (for a review, see Matute et al., 2015). The illusion of causality has previously been associated with the development and maintenance of pseudomedicine beliefs (Matute, Yarritu, & Vadillo, 2011), as well as judgements of guilt in a criminal setting (Lassiter, Geers, Munhall, Ploutz-Snyder, & Breitenbecher, 2002). We argue that this cognitive bias also presents a problem to educators, as it might result in teachers endorsing teaching practices that are not effective in improving students’ academic performance. We, of course, do not suggest that all false beliefs that teachers hold are the result of observational contingency learning in the classroom or that contingency learning is the only mechanism that reinforces such beliefs; e.g. it would be difficult to see how some false beliefs, such as the belief that we only use 10% of our brains (endorsed by nearly 50% of teachers; Dekker et al., 2012), are perpetuated through contingency learning in the classroom. However, many beliefs that teachers hold are about the efficacy of their own practices which presumably are based on causal inferences about the impact of their teaching practices, which may be driven by contingency learning.

## Outcome densities and causal inference

The ability to correctly estimate the contingency between two events relies on an accurate memory of the outcome occurring in the presence and absence of the cue. Experimental research on illusory causation effects has explored the frequency of cue and outcome events as potential factors that inflate false causal beliefs, where manipulations that increase cue and outcome coincidences are particularly effective in biasing strong false beliefs (e.g. Wasserman, 1990). One pertinent example of this is the outcome density effect (Blanco & Matute, 2015; Chow et al., 2019). The outcome density effect is the tendency for people to overestimate the causal relationship between a cue and an outcome when the base rate of the outcome occurring is high relative to when the base rate is low, even when the outcome is independent of the cue. The outcome density effect has been reliably produced using binary outcome events (Blanco & Matute, 2015), when the outcome event is variable (non-discrete) and ambiguous in relation to the participant’s putative causal belief (Chow et al., 2019), when the cue-outcome events are presented one trial a day across a 24-day time scale (Willett & Rottman, 2019), and when the genuine contingency is negative (Vallée-Tourangeau, Murphy, & Baker, 2005; Wasserman, Elek, Chatlosh, & Baker, 1993). In a classroom setting, the outcome density effect may play a role in biasing teachers’ ability to accurately determine the effectiveness of their teaching practices if the student cohort is high-achieving and, therefore, likely to perform well academically regardless of the teaching practice used. Some researchers have also proposed that the development of strong false beliefs are able to interfere with subsequent acquisition of real causal relationships (Yarritu, Matute, & Luque, 2015), suggesting that these beliefs may be persistent and difficult to correct.

Although we will not be manipulating the frequency of the cue in this series of experiments, it is important to note that a high frequency of cue-present trials (e.g. implementing the teaching practice regularly) also result in heightened illusory causation relative to when the cue is presented infrequently. This is referred to as the cue density bias (Allan & Jenkins, 1983; Matute et al., 2011). In theory, these event densities may present a cycle of illusory belief that is difficult to break: teachers develop strong false belief in the efficacy of an ineffectual teaching practice when they have a high-performing cohort (i.e. the outcome density effect), and this results in the persistence of the teaching practice that further strengthens the belief in its efficacy (i.e. the cue density effect), although neither the outcome or cue density effect have been shown in educationally relevant situations previously. Therefore, it is pertinent that we examine illusory causation and the outcome density effect, in particular, in an educational context to determine the extent to which students’ academic outcomes influence people’s belief in a novel teaching practice that is objectively ineffective (Experiments 1–3, 5, and 6) and even detrimental to student performance (Experiment 4).

## Outcome densities in educational assessments

Classroom-based assessments are often used by teachers to gauge students’ achievement and learning. Teachers use these assessments to gauge whether their instruction and teaching methods are working, and often adjust their practices according to their students’ results on assessments (Pellegrino, Chudowsky, & Glaser, 2001). Teachers typically infer the effectiveness of their practice by considering the contingency between their practice and the aggregate level of performance due to the computational complexity and memory demands of basing their inferences on student-level data (Black & Wiliam, 2018; Fiedler, Freytag, & Meiser, 2009). For example, Fiedler, Freytag, and Unkelbach (2007) found that teachers gathering observations to infer correlations between student beliefs in a simulated classroom environment were biased by contingencies at the aggregate (i.e. classroom) level. This raises the question of whether the distribution of outcomes of a teacher’s class can influence their ability to make accurate causal inferences about the efficacy of their practices.

In practical terms, classroom assessments are typically designed so that performance is relatively high. This is typically done out of a desire to allow all students to ‘show what they know’ as well as to promote and protect students’ self-efficacy (e.g. Kang, Thompson, & Windschitl, 2014; McCabe, 2003). It is, therefore, worth noting that the distribution of most classrooms assessments will, in practice, have a high base rate with frequent positive outcomes. The real-world context of classroom assessments thus appear to be analogous to a typical high outcome density condition used in contingency learning experiments, a condition where causal illusions are significantly more likely (Matute et al., 2015). If teachers use classroom assessments to guide and evaluate their practice then designing classroom assessments such that a vast majority of students perform well may, in fact, be biasing teachers to believe in the efficacy of ineffectual practices as well as limiting the utility of classroom assessments as a source of feedback for teachers.

## Current study

The current study examines whether the distribution of students’ academic outcomes affects the inferences that an observer makes about the efficacy of a novel instructional technique. Adapting the typical contingency learning paradigm for an educational context and examining both categorical and continuous outcomes, we aim to examine whether frequent positive student outcomes promote false beliefs. In Experiment 1, we first examine whether an outcome density effect is observed in the context of participants’ instructional beliefs when students’ test performance is presented as a discrete outcome (high vs. low performance). A discrete outcome format is typically used within the outcome density literature (Chow et al., 2019). In Experiments 2 and 3, we explore whether outcome density affects participants’ instructional beliefs when test performance is presented as a continuous outcome, using either a skewed (Experiment 2) or normal (Experiment 3) distribution of outcomes. In Experiment 4 we examine whether inaccurate beliefs about the efficacy of a novel instructional technique persist when there is a negative contingency between the use of the novel teaching technique and student outcomes. In Experiment 5, we examine the outcome density effect when student outcomes are presented concurrently as they might in a classroom environment, and, finally in Experiment 6 we replicate the basic outcome density finding in a sample of teachers.

## Experiment 1

### Participants

Participants were recruited using Amazon’s Mechanical Turk (Mturk). Participation was restricted to participants from the US who had at least a 95% approval rate on the site. Participants were paid 80 cents for participation in the study. A power analysis suggested that a minimum sample size of 78 participants was required to detect an effect size of .65 with 80% power. We recruited a total of 80 (48% female) participants to account for the possibility that data would need to be discarded. Forty-one participants were randomly allocated by computer to the high outcome density (High-OD) condition and 39 participants were allocated the low outcome density (Low-OD) condition. The average age of the participants was 37.08 (standard deviation (SD) = 9.53).

### Material and procedure

Before commencing the experiment, participants were told to imagine that they were a school teacher trialling a new instructional method called ‘Kalavatic teaching’. Participants were told that each student in their class had either been taught with this novel method or an ‘ordinary teaching’ method, and it was their goal to determine whether the new teaching method was effective at improving performance in an examination. Participants were told that this novel method of teaching was hypothesised to improve memory and academic performance in students.

**Fig. 1:**
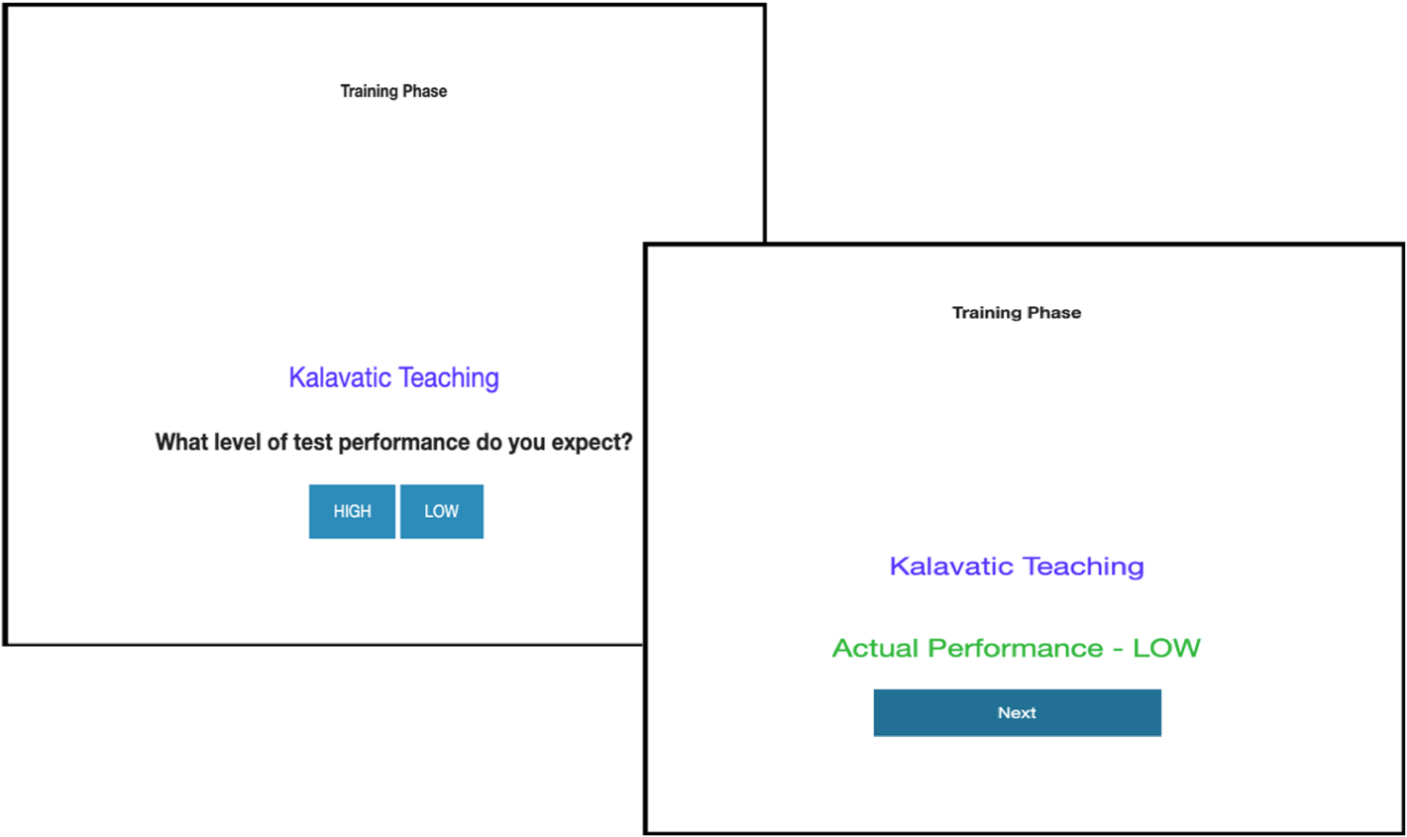
Example training trial from Experiment 1

Participants then performed a contingency learning task that consisted of a training and test phase. Each trial during the training phase represented one student in the class. For each trial, participants observed whether Kalavatic teaching (i.e. cue-present) or ordinary teaching (i.e. cue-absent) was administered to that particular student. Participants were then asked to predict the student’s performance in the examination. Finally, after making their prediction, the participant was shown an outcome that was ostensibly the student’s actual performance in the examination (see Fig. 1). There were 30 trials in the training phase. This number of trials was selected because it maps onto typical class sizes in the US. Cue presence/absence was randomly determined for each trial and each participant such that the cue was present on 50% of trials in the training phase.

For Experiment 1, the outcome (and prediction) was binary with students either having ‘high’ or ‘low’ performance in the examination. Cue presence and absence was distinguished by presenting the teaching method used in large blue font on each trial, either Kalavatic teaching on cue-present trials or ordinary teaching on cue-absent trials. The prediction was accompanied by the instruction ‘What level of test performance do you expect?’. Participants indicated their prediction by clicking a button with their mouse (either ‘*high*’ or ‘*low*’). After making their prediction, the outcome was displayed in the format ‘Actual Performance – *high* [*low*]’ in green font. Each trial was participant paced, such that they clicked ‘Next’ when they were ready to progress to the next trial.

**Fig. 2:**
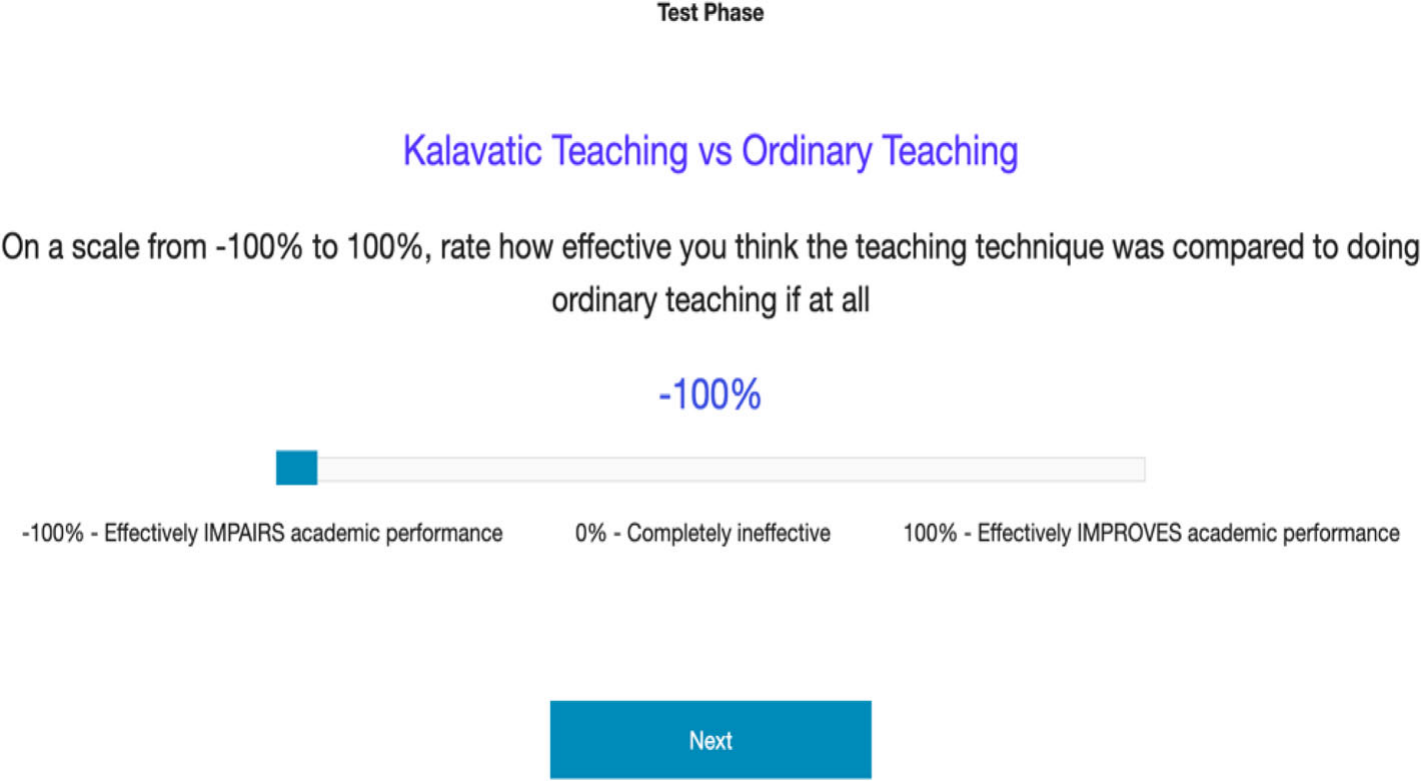
Example test trial from Experiments 1–6

During the test phase, participants were asked to make a causal rating by judging how effective Kalavatic teaching was compared to ordinary teaching on a scale ranging from − 100 (‘Effectively IMPAIRS academic performance’) to 100 (‘Effectively IMPROVES academic performance’). The midpoint (zero) was labelled with the anchor (‘Completely ineffective’). The question asked ‘On a scale from − 100 to 100%, rate how effective you think the teaching technique was compared to doing ordinary teaching, if at all’. At the top of the page there was a heading: ‘Kalavatic teaching vs. ordinary teaching’ (see Fig. 2). The following note was also provided below the rating to participants while they made their judgement:

‘*Note that intermediate negative values indicate the teaching technique actually makes academic performance worse whereas intermediate positive values indicate that the teaching technique was effective in improving academic performance*’*.*

### Design

Participants were randomly allocated to either a high outcome density condition or a low outcome density condition, the difference being the ratio of outcomes. For the High-OD condition 20/30 of students had ‘high’ performance in the examination (10/30 ‘low’), while for the Low-OD condition 10/30 had ‘high’ performance on the examination (20/30 ‘low’). The outcome for each trial (high/low) was presented in a random intermixed fashion from the specified distributions. In both conditions there was no contingency between the presentation of the cue (Kalavatic teaching vs. ordinary teaching) and the outcome (high vs. low; *Δp* = 0).

### General analytical approach

In each experiment we compare the high and low outcome density conditions on each dependent variables separately. Comparisons are made using both Bayes Factors and Frequentist statistics. Where reported, the BF_10_ is the likelihood of the alternative model compared to the null model given the data, where the null model specifies no difference between groups. For the between-within-subjects analyses of variance (ANOVAs), we report the Bayes Factor Inclusion/Exclusion (BF_inc_/BF_excl_) across matched models (Rouder, Morey, Verhagen, Swagman, & Wagenmakers, 2017), which indicates the evidence that a model including the interaction term is a better fit for the data compared to an equivalent model without an interaction term.

## Results and discussion

### Training data

**Fig. 3:**
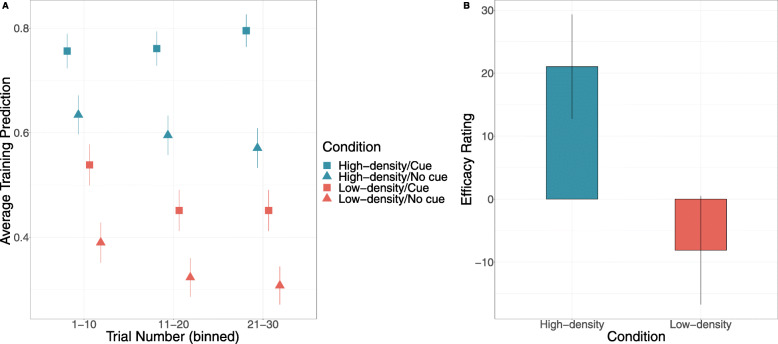
Results from Experiment 1 for **a** training predictions as a function of outcome density condition, cue presence, and trial as well as **b** efficacy ratings as a function of outcome density condition. Error bars represent +1 standard error of the mean

We begin by examining participants’ predictions made throughout training. Of primary interest is whether outcome density condition interacts with cue presence. A 2 (Kalavatic teaching vs. ordinary teaching) X 2 (high vs. low outcome density) between-within-subject ANOVA was performed, with the proportion of ‘high’ predictions used as the dependent variable. As shown in Fig. 3a, participants made substantially more ‘high’ predictions of the student’s performance when they were taught with Kalavatic teaching (*M* = .63, *SD* = .26) compared with when they were taught with ordinary teaching (*M* = .47, *SD* = .27), *F* = 15.77, *p* < .001, *η*_*p*_^2^ = .17, BF_10_ > 100. Furthermore, participants made more ‘high’ predictions on average when they were in the high outcome density group (*M* = .69, *SD* = .22) compared to the low outcome density group (*M* = .41, *SD* = .25), *F* = 79.82, *p* < .001, *η*_*p*_^2^ = .51, BF_10_ > 100. Crucially, however, the interaction between cue presence and outcome density condition was not significant, *F* = 0.15, *p* = .70, η_p_^2^ = .002, BF_inc_ = .248. These results suggests that outcome density did not affect participants predictions of the effect of Kalavatic teaching relative to ordinary teaching in training. This finding is consistent with previous research and may suggest important differences between the processes involved in making predictions in training compared to a making causal judgements (Blanco & Matute, 2015; Chow et al., 2019). This finding will be discussed in greater detail in the general discussion.

### Efficacy ratings

Next, we examined whether there was an outcome density effect with respect to participants’ causal ratings. As shown in Fig. 3b, participants in the high outcome density condition (*M* = 21.02, *SD* = 53.03) made significantly higher efficacy ratings compared to participants in the low outcome density condition (*M* = − 8.13, *SD* =53.75, *t* (78) = 2.44, *p* = .017, *d* = .55, BF_10_ = 2.95). The efficacy ratings for participants in the high outcome density group were significantly higher than zero, *t* (40) = 2.54, *p* = .015, *d* = .40, BF_10_ = 2.84, while the efficacy ratings for the low outcome density group were not, *t* (38) = .94, *p* = .351, *d* = − .15, BF_10_ = .26.

The results of Experiment 1 suggest that the frequency of assessment outcomes affects the causal efficacy ratings made by participants with respect to a novel instructional technique. This finding replicates the classic outcome density effect that has typically been observed in causal or efficacy ratings in other learning contexts. The result provides an in-principle demonstration that similar contingency learning biases may affect judgements of educational effectiveness in the classroom. Participants observing frequent positive outcomes (high performance) appear to be more susceptible to incorrectly inferring that the novel teaching treatment is effective.

## Experiment 2

While Experiment 1 established an outcome density effect on causal beliefs about instructional techniques using the discrete paradigm typical of contingency learning studies, such discrete outcomes are less frequent in the classroom. Most classroom-based assessments and exams are marked on a continuous scale (although some categorisation is also typical, e.g. A+). We therefore now turn in Experiment 2 to examining whether teaching beliefs are affected by the frequency of assessment outcomes when such outcomes are presented using a continuous scale.

The outcome density effect has recently been applied to continuous outcomes by Chow et al. (2019). They found that in a health context, continuous outcomes still produced illusory causation and outcome density effects. They used two types of distribution to test this. In Experiment 1, they used a bimodal distribution combining two normal distributions, one centred on high outcome values, one centred on low outcome values, varying the proportion of trials sampled from each distribution to create High-OD and Low-OD conditions. In Experiment 2, they used unimodal distributions that were either centred on a low value and positively skewed or centred on a high value and negatively skewed. Here in Experiment 2 we similarly utilise skewed unimodal distributions, before using symmetrical normal distributions in Experiment 3.

## Method

### Participants

Participants were recruited in the same fashion as Experiment 1. 80 (45% female) participants were recruited and randomly allocated to condition by computer (*n* = 44 High-OD, *n* = 36 Low-OD). The average age of the participants was 39.1 (*SD* = 10.66).

### Materials and procedure

**Fig. 4:**
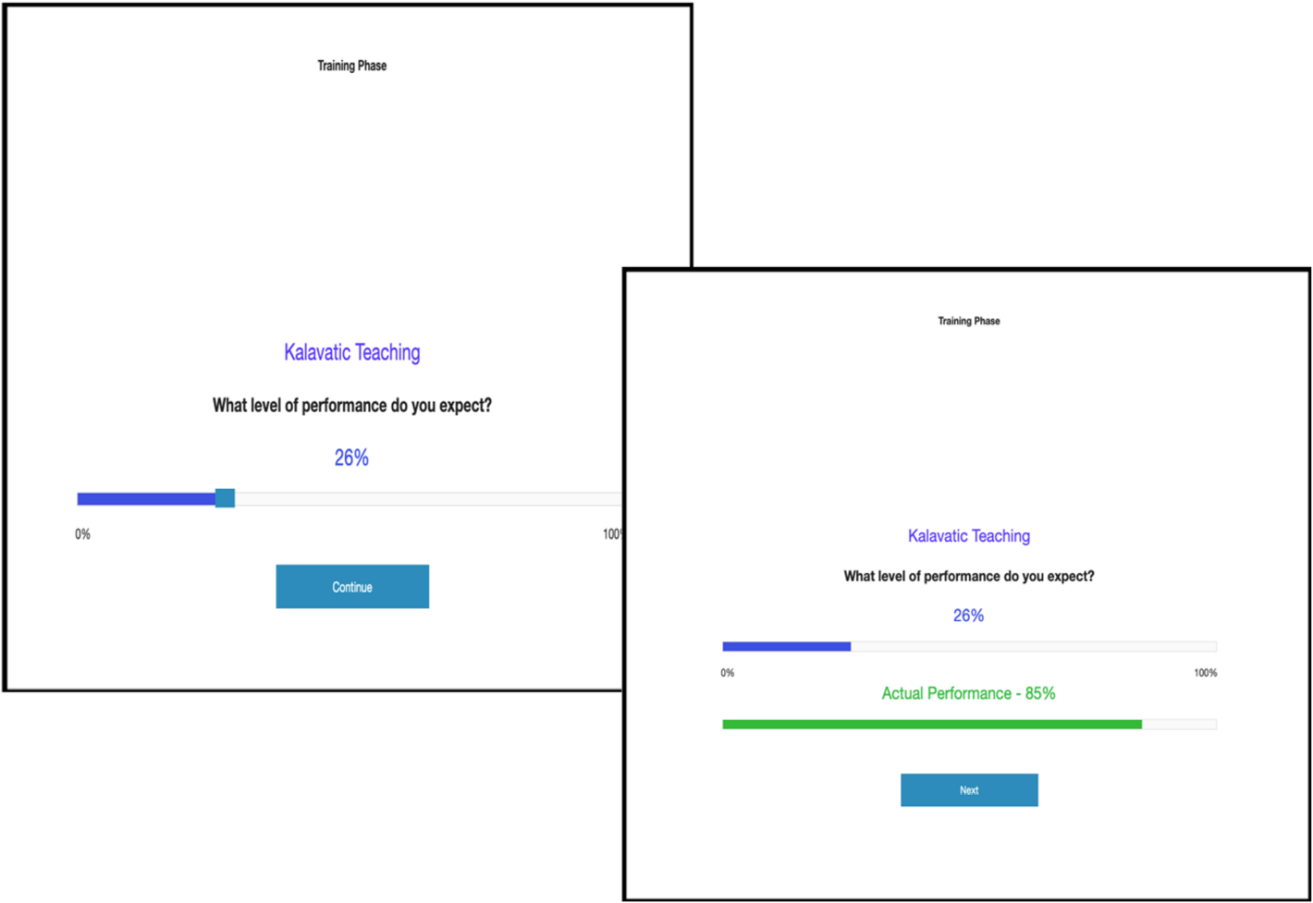
Example trial from Experiments 2–4 and Experiment 6

The procedure used in Experiment 2 was similar to that used in Experiment 1, except that the outcomes (the students’ examination performance) were continuous. Predictions during training were made along a visual analogue scale ranging from 0 to 100% (see Fig. 4). After participants made their prediction, the outcome was displayed on screen using the same visual scale.

Additionally, during the test phase in Experiment 2, after making the causal rating, participants were asked to predict the average performance of students who were taught with Kalavatic teaching and the average performance of students taught with ordinary teaching. This measure was included to determine whether average predictions made at test would still show reliable illusory causation effect—that is, participants predict greater performance for students given Kalavatic teaching than those given ordinary teaching despite overall base rate of performance being identical for cue-present and cue-absent trials—and whether differences in prediction are greater for High-OD relative to Low-OD participants, indicative of an outcome density effect. Based on findings from Chow et al. (2019), outcome density effects are most reliably produced in causal judgements, with little evidence of the effect in prediction judgements (see also Vadillo, Miller, & Matute, 2005 on the dissociation between causal and prediction judgements). Thus, we did not anticipate an interaction between cue type and outcome condition (indicative of an outcome density effect) on average prediction ratings, however, we expected participants to show reliable illusory causation by predicting greater performance with Kalavatic teaching than ordinary teaching.

### Design

**Fig. 5:**
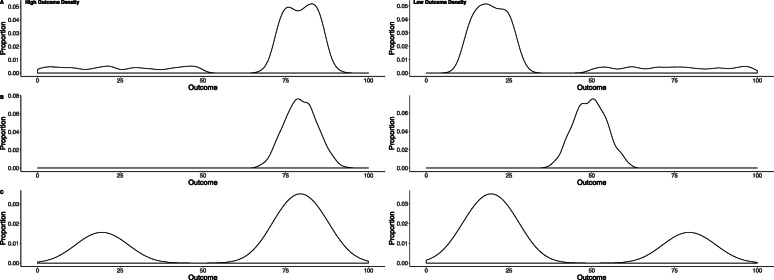
Example outcome distributions used in **a** Experiment 2, **b** Experiments 3, 5, and 6 and **c** Experiment 4. Distributions for the high outcome density condition are the left panels, while distributions for the low outcome density condition are the right panels

Outcomes were randomly sampled for each participant from a distribution described below and presented in Fig. 5a. We opted to utilise the same proportion of trials from the high/low distributions as Chow et al. (2019). For participants in the High-OD condition 80% of outcomes were stipulated to be from a distribution with a mean of 80%, a SD of 5%, and a range of 50–100%. The remaining 20% of trials were sampled randomly from values ranging from 0 to 50%. For participants in the Low-OD condition, 80% of outcomes were sampled from a distribution with a mean of 20%, a SD of 5%, and a range of 0–50%. The remaining 20% of trials were sampled randomly from values ranging from 50 to 100%.

## Results and discussion

### Training data

**Fig. 6:**
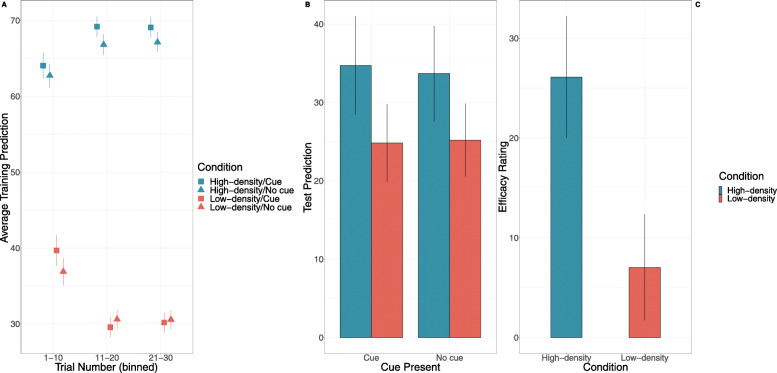
Results from Experiment 2 for **a** training predictions as a function of outcome density condition, cue presence, and trial as well as **b** predictions of students’ average performance as a function of outcome density condition and **c** efficacy ratings as a function of outcome density condition. Error bars represent + 1 standard error of the mean

Again, a 2 (Kalavatic teaching vs. ordinary teaching) X 2 (high vs. low outcome density) between-within-subject ANOVA was performed. Depicted in Fig. 6a, the results suggested that participants did not make significantly different predictions when the cue was present, that is, Kalavatic teaching was administered (*M* = 51.99, *SD* =21.22) compared to when it was absent (*M* = 50.76, *SD* = 20.23; *F* = 1.04, *p* = .310, *η*_*p*_^2^ = .01, BF_10_ = .29). The High-OD condition (*M* = 66.48, *SD* = 13.42) made significantly higher predictions than the Low-OD density condition (*M* = 32.9, *SD* = 10.38), *F* = 181.28, *p* < .001, *η*_*p*_^2^ = .70, BF_10_ > 100. Most importantly, outcome density condition did not interact with the presence of the cue *F* = .39, *p* = .53, *η*_*p*_^2^ = .005, BF_inc_ = .27. As with Experiment 1, this suggests that there was no outcome density effect on the predictions made during training.

### Aggregate predictions

Finally, we examined whether outcome condition affected participants’ predictions of the average performance of students who were taught with Kalavatic or ordinary teaching. A 2 (Kalavatic teaching vs. ordinary teaching) X 2 (high vs. low outcome density) within-between-subjects ANOVA was run. The results suggested that there was no difference in predictions at test between the high (*M* = 34.19, *SD* = 28.85) and low (*M* = 25.01, *SD* = 20.23) outcome conditions, *F* = 3.14, *p* = .08, *η*_*p*_^2^ = .04, BF_10_ = 1.08, nor was there any difference in test predictions between Kalavatic teaching (*M* = 30.26, *SD* = 26.34) and ordinary teaching (*M* = 29.86, *SD* = 25.16), *F* = .02, *p* = .89, *η*_*p*_^2^ < .001, BF_10_ = .18. Finally, the interaction between cue type and outcome density condition was not significant, *F* = .08, *p* = .78, *η*_*p*_^2^ = .001, BF_inc_ = .24 (see Fig. 6b).

### Efficacy ratings

As shown in Fig. 6c, participants in the High-OD condition (*M* = 26.11, *SD* = 40.39) made significantly greater efficacy ratings than participants in the Low-OD condition (*M* = 7.03, *SD* = 31.76), *t* (78) = 2.31, *p* = .024, *d* = .52,BF_10_ = 2.27). Participants in the High-OD condition made efficacy rating significantly higher than zero, *t* (43) = 4.29, *p* < .001, *d* = .65, BF_10_ = 237.69, whereas participants in the Low-OD condition did not, *t* (35) = 1.33, *p* = .193, *d* = .17 BF_10_ = .40. These findings suggest that illusory causation and outcome density effects are most prevalent in efficacy ratings and not when participants are asked to make a predictive judgement.

In addition, because the methodology in Experiments 2–6 requires random sampling from a distribution, the precise contingency that participants observe may differ slightly by chance. To account for this we re-ran all models in Experiments 2–6 with the observed contingency (measured as the mean difference between cue-present/-absent trials observed outcomes) as a covariate. None of the results changes substantively.

## Experiment 3

The results of Experiment 2 largely replicate those of Experiment 1 in suggesting that the frequency of assessment outcomes affects efficacy ratings of instructional practices. Again, participants who observed frequent positive outcomes (high student performance) incorrectly rated the novel instructional technique as effective. Importantly these results suggest that even when assessment outcomes are conveyed on a continuous scale, participants are prone to an outcome density effect.

The distributions in Experiment 2 were effectively skewed, such that almost all participants performed well in the High-OD group or poorly in the Low-OD group. Participants could, therefore, essentially partition these outcomes categorically into one of two outcomes, despite the added variability of a continuous outcome. While the skewed distributions map closely onto traditional outcome density paradigms and the continuous outcome distributions used in Chow et al. (2019), it remains to be seen whether outcome density effects can be readily observed with normally distributed outcomes. This is important because assessment results are generally normally distributed and, to our knowledge, the outcome density effect has never been shown using normally distributed outcomes. In Experiment 3 we therefore replicated Experiment 2, but this time used two normally distributed outcome distributions where participants would be less able to classify students’ performance into discrete categories.

## Method

### Participants

Eighty participants (56% female) were recruited in the same fashion as in the previous experiments (*M*_*age* =_ 38.42, *SD* = 11.83). Forty participants were randomly allocated to the High-OD condition and 40 participants to the Low-OD condition.

### Procedure and design

The same procedure and design as in Experiment 2 was used, except that the distribution of assessment outcomes differed (see Fig. 5b). For the High-OD condition, all of the outcomes that participants observed were randomly drawn from a normal distribution with a mean of 80 and a SD of 5. Whereas for the Low-OD condition, all of the outcomes were drawn from a normal distribution with a mean of 50 and a SD of 5. Test ratings remained unchanged.

## Results and discussion

### Training data

**Fig. 7:**
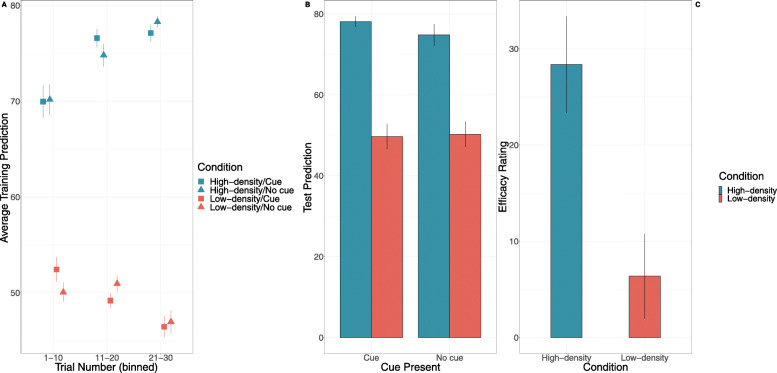
Results from Experiment 3 for **a** training predictions as a function of outcome density condition, cue presence, and trial as well as **b** predictions of students’ average performance as a function of outcome density condition and **c** efficacy ratings as a function of outcome density condition. Error bars represent + 1 standard error of the mean

A 2 (Kalavatic teaching vs. ordinary teaching) X 2 (high vs. low outcome density) ANOVA indicated that participants did not make significantly different predictions when the cue was present (*M* = 61.95, *SD* = 15.97) compared to when it was absent (*M* = 61.87, *SD* = 15.96; *F* = .03, *p* = .86, *η*_*p*_^2^ < .001, BF_10_ = .18) (see Fig. 7a). The High-OD condition (*M* = 74.48, *SD* = 9.49) made significantly higher predictions than the Low-OD condition (*M* = 49.34, *SD* = 9.98), *F* = 138.25, *p* < .001, *η*_*p*_^2^ = .64, BF_10_ > 100. Consistent with the previous results, outcome density condition did not interact with the presence of the cue *F* = .01, *p* = .92, *η*_*p*_^2^ < .001, BF_inc_ = .22.

### Aggregate predictions

A 2 (Kalavatic teaching vs. ordinary teaching) X 2 (high vs. low outcome density) within-between-subjects ANOVA was run to examine whether there were group differences in the predictions of average performance at test. Results indicated that High-OD (*M* = 76.46, *SD* = 9.52) condition made significantly higher predictions of average performance compared to the Low-OD (*M* = 49.96, *SD* = 13.77) condition, *F* = 140.40, *p* < .001, *η*_*p*_^2^ = .64, BF_10_ > 100 (see Fig. 7b). More importantly, there was again no significant difference in predictions of average performance between Kalavatic teaching (*M* = 63.89, *SD* = 17.82) and ordinary teaching (*M* = 62.54, *SD* = 17.82), *F* = .90, *p* = .35, *η*_*p*_^2^ = .01, BF_10_ = .25, i.e. no illusory causation effect. Finally, the interaction between cue type and outcome density condition was not significant, *F* = 1.83, *p* = .18, *η*_*p*_^2^ = .02, BF_inc_ = .53.

### Efficacy ratings

Participants in the High-OD (*M* = 28.38, *SD* = 31.67) condition made significantly greater efficacy ratings than participants in the Low-OD (*M* = 6.38, *SD* = 27.9) density condition, *t* (78) = 3.30, *p* = .001, *d* = .74, BF_10_ = 21.84) (see Fig. 7c). Again, the High-OD group made efficacy ratings significantly higher than zero, *t* (39) = 5.67, *p* < .001, *d* = .90, BF_10_ > 100, whereas the Low-OD group did not, *t* (39) = 1.45, *p* = .156, *d* = .20, BF_10_ = .45.

The results of Experiment 3, in particular the efficacy ratings, confirm that participants who observe frequent high-performing students are more likely to infer that a novel instructional technique being trialled is effective. These findings suggest that an outcome density effect can be observed even when a symmetrical normal distribution is used. This is particularly noteworthy because if participants were to categorise their students’ performance into discrete relative categories (e.g. higher than average, lower than average), then the frequencies of these events would be identical in the Low-OD and High-OD conditions. Instead, the outcomes are only disproportionately high in the High-OD condition relative to their framing on the scale provided, nothing else. To our knowledge all other demonstrations of the outcome density effect use frequency differences between outcomes that are high and low relative to the range of outcomes that are actually experienced; for instance, the outcome takes one of two values (present/absent or high/low), one of which is more frequent than the other. These findings suggest that a causal illusion can arise even when participants do not observe biased frequencies of outcomes that are relatively high and relatively low, suggesting that the framing of the scale—and perhaps pre-existing beliefs about what constitutes a ‘good’ or ‘bad’ score on an assessment—are used to evaluate whether an outcome is favourable.

## Experiment 4

While all of the experiments thus far have shown that efficacy ratings are affected by the distribution of assessment outcomes, they have all been under conditions where there is no effect of the cue on the outcome (*Δp* = 0). In Experiment 4 we extend these findings by examining whether an outcome density effect leads to false beliefs about the efficacy of an instructional technique when it has a *negative* effect on student outcomes (*Δp* = − .2). Outcome density effects have previously been shown when the effect of the cue on the outcome is negative (Vallée-Tourangeau et al., 2005; Wasserman et al., 1993). In this instance, a rational observer should be judging that the novel practice is effectively producing *worse* performance compared to regular instruction. If the base rate of high achievement observed in under High-OD conditions is sufficient to still produce significant positive ratings of the novel practice, then this is particularly concerning.

## Method

### Participants

Eighty participants (60% female*; M*_*age* =_ 42.32, *SD* = 12.22) were recruited in the same fashion as in the previous experiments (*n* = 41 High-OD; *n* = 39 Low-OD).

### Procedure and design

The same design as the previous experiments was used except for the distributions and contingency. In order to calculate a contingency utilising *Δp*, we sampled from two distributions (a high-magnitude and a low-magnitude distribution) for each condition, but at different rates. The high-magnitude distribution had a mean of 80 and the low-magnitude distribution had a mean of 20 (both *SD* = 5). For the High-OD condition, 70% of outcomes were randomly sampled from the high-magnitude distribution and 30% of outcomes were sampled from the low-magnitude distribution averaged over cue presence (see Table 1 for a breakdown by cue presence). For participants in the Low-OD condition, 70% of outcomes were randomly sampled from the low-magnitude distribution while 30% of outcomes were sampled from the high-magnitude distribution averaged over cue presence. Distributions are presented in Fig. 5c.

**Table 1:** Outcome contingencies used in Experiment 4

	High-OD		Low-OD	
	High-magnitude	Low-magnitude	High-magnitude	Low-magnitude
Novel teaching	0.3	0.2	0.1	0.4
Ordinary teaching	0.4	0.1	0.2	0.3

Note: The low-magnitude distribution had a mean of 20, the high-magnitude distribution had a mean of 80 (both *SD* = 5). Cells represent the number of trial from each sample and whether the cue was present (*Δp* = 0)

*OD* outcome density

As shown in Table 1, a negative contingency between the cue (Kalavatic teaching) and the outcome was set. *Δp* was calculated using the ratio of outcomes drawn from the high-magnitude relative to the low-magnitude distribution and was equal to − .20.

## Results and discussion

### Training data

**Fig. 8:**
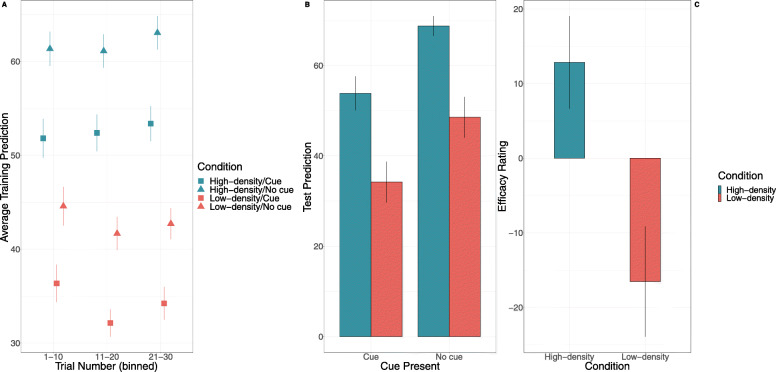
Results from Experiment 4 for **a** training predictions as a function of outcome density condition, cue presence, and trial as well as **b** predictions of students’ average performance as a function of outcome density condition and **c** efficacy ratings as a function of outcome density condition. Error bars represent + 1 standard error of the mean

Participants make significantly lower predictions when the cue was present (*M* = 43.61, *SD* = 18.36) compared to when it was absent (*M* = 52.64, *SD* = 17.82; *F* = 29.65, *p* < .001, *η*_*p*_^2^ = .28, BF_10_ > 100), suggesting a sensitivity to the negative contingency between cue-present trials and student performance (see Fig. 8a). The High-OD condition (*M* = 57.18, *SD* = 18.29) made significantly higher predictions than the low outcome (*M* = 38.61, *SD* = 13.55) density condition, *F* = 36.55, *p* < .001, *η*_*p*_^2^ = .32, BF_10_ > 100. However, outcome density condition did not interact with the presence of the cue *F* = .03, *p* = .87, *η*_*p*_^2^ < .001, BF_inc_ = .23.

### Aggregate predictions

As with Experiment 3, the results indicated that High-OD (*M* = 61.28, *SD* = 15.71) condition made significantly higher predictions of average performance compared to the Low-OD (*M* = 41.37, *SD* = 21.08) condition, *F* = 58.16, *p* < .001, *η*_*p*_^2^ = .43, BF_10_ > 100. Furthermore, participants made lower predictions of average performance for Kalavatic teaching (*M* = 44.26, *SD* = 20.85) compared to ordinary teaching (*M* = 58.89, *SD* = 18.54), *F* = 27.31, *p* < .001, η_p_^2^ = .26, BF_10_ > 100. Finally, the interaction between cue type and outcome density condition was not significant, *F* = .01, *p* = .92, *η*_*p*_^2^ < .001, BF_inc_ = .22 (see Fig. 8b).

### Efficacy ratings

Participants in the High-OD (*M* = 12.83, *SD* = 39.72) condition made significantly greater efficacy ratings than participants in the Low-OD (*M* = − 16.54, *SD* = 46.11) condition, *t* (78) = 3.06, *p* = .003, *d* = .68, BF_10_ = 11.82) (see Fig. 8c). Participants in the High-OD condition made efficacy ratings significantly higher than zero, *t* (40) = 2.07, *p* = .045, *d* = .32, BF_10_ = 2.07, while participants in the Low-OD condition made ratings significantly lower than zero, *t* (38) = 2.24, *p* = .031, *d* = − .42, BF_10_ = − .30. This suggests that the negative contingency between cue and outcome was sufficiently large enough to be detected by participants in the low outcome density condition; however, despite this, participants in the high outcome condition still believed that the novel teaching technique was beneficial.

## Experiment 5

The previous studies have shown that participants’ beliefs about the efficacy of a novel teaching technique are susceptible to an outcome density effect across a range of distributions and contingencies. These experiments have, however, all presented student outcomes sequentially. While many assessment outcomes will be observed by teachers in this fashion; e.g. grading essays or marking exams one after another, student outcomes will also sometimes be received concurrently. A teacher may, for example, try out a new teaching technique and then walk around the class observing how her students are performing on a task. In order to examine whether outcome density effects can similarly occur under such plausible classroom scenarios, Experiment 5 presents the outcomes to participants in groups of nine students, with only three trials per cue.

## Method

### Participants

Eighty participants (*M* = 37.84, *SD* = 11.34) were recruited in the same fashion as the previous experiments (41% female). Thirty-nine participants were randomly allocated to the High-OD condition and 41 were allocated to the Low-OD condition. The data from 10 participants was excluded from the analysis because they showed signs of random responding (all completed the study in under 60 s).

### Procedure and design

**Fig. 9:**
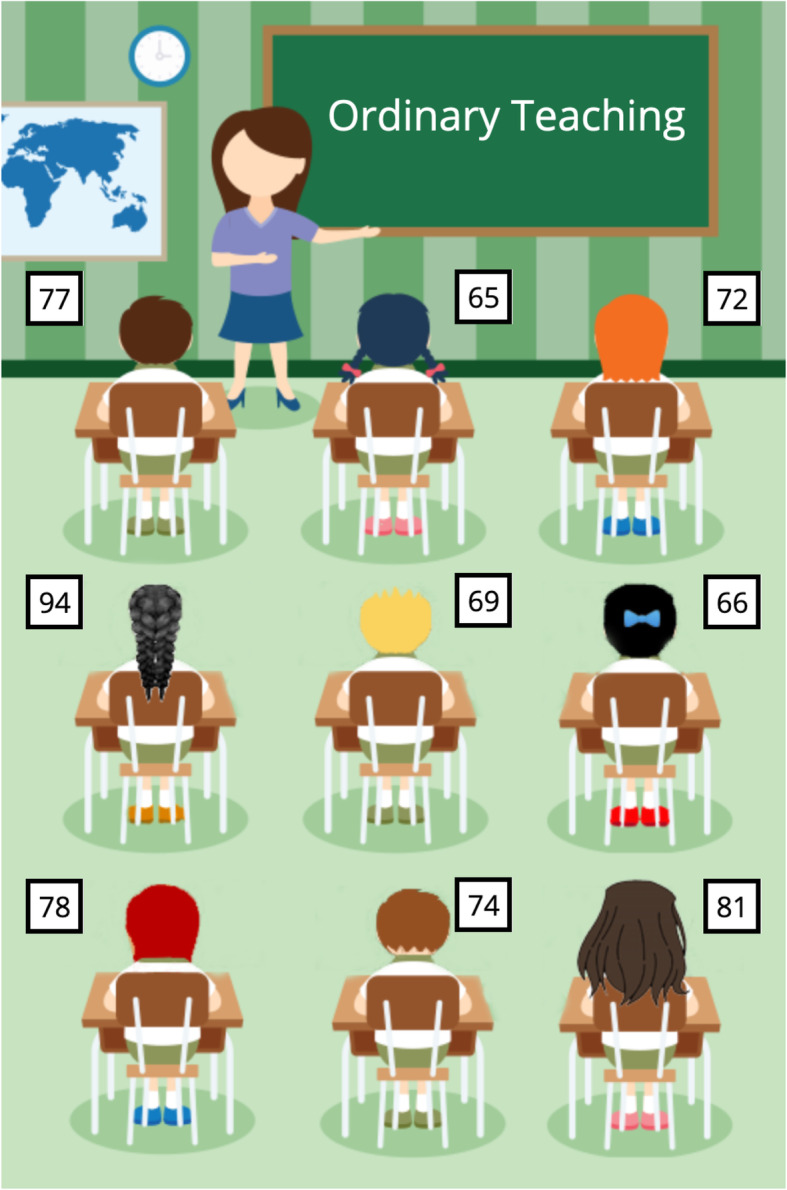
An example of a no-cue trial from Experiment 5 showing the simulated classroom outcomes

The distributions from Experiment 3 were utilised (as symmetrical distributions were deemed to be the most educationally realistic), with outcomes in the High-OD having a mean of 80 (*SD* = 5) and the outcomes in the Low-OD condition having a mean of 50 (*SD* = 5). Outcomes were presented for nine students at a time in three rows of three using a classroom backdrop (see Fig. 9). The teaching strategy (Kalavatic teaching, ordinary teaching) was shown on the ‘blackboard’ indicating cue presence/absence. Participants were shown six trials (three cue and three no cue) in a randomised order, for a total of 27 outcomes for each cue. Participants made no predictions during training, instead they simply pressed ‘Next’ to proceed to the next trial. At the conclusion of the six training trials participants made aggregate predictions and an efficacy rating in the same manner as the previous experiments.

## Results and discussion

### Aggregate predictions

**Fig. 10:**
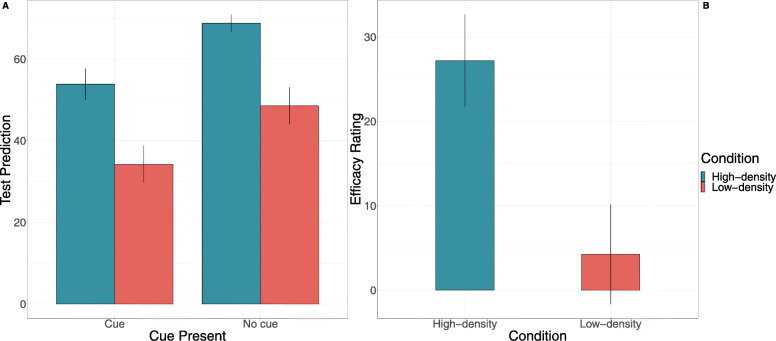
Results from Experiment 5 for **a** predictions of students’ average performance as a function of outcome density condition and **b** efficacy ratings as a function of outcome density condition. Error bars represent + 1 standard error of the mean

Participants in the High-OD condition (*M* = 68.56, *SD* = 25.82) made significantly higher aggregate predictions compared to participants in the Low-OD condition, *M* = 52.67, *SD* = 17.95; *F* = 11.90, *p* = .001, *η*_*p*_^2^ = .15, BF_10_ = 32.49, averaged across cue presence. There was no significant difference between aggregate predictions for Kalavatic teaching (*M* = 57.69, *SD* = 25.35) compared to ordinary teaching *M* = 61.73, *SD* = 20.62; *F* = 2.62, *p* = .110, *η*_*p*_^2^ = .04, BF_10_ = .59, nor was the interaction between outcome density condition and cue presence significant, *F* = .03, *p* = .858, *η*_*p*_^2^ < .001, BF_inc_ = .24 (see Fig. 10a).

### Efficacy ratings

Participants in the High-OD condition (*M* = 27.19, *SD* = 30.47) again made significantly higher efficacy ratings than the Low-OD condition: *t* (68) = 2.79, *p* = .007, *d* = .67, BF_10_ = 6.25 (see Fig. 10b).

## Experiment 6

While the previous experiments have consistently shown an outcome density effect using educationally relevant material, each has been performed on a community sample. This raises the question of whether teachers are equally susceptible to the outcome density effect. While there is no strong theoretical reason to suppose that teachers will vary substantially from the general population in this regard, teachers may have strong prior beliefs about the likely efficacy of novel teaching techniques or may be better able to assess the efficacy of teaching techniques due to the necessity of such inferences in their profession. In Experiment 6, we replicate Experiment 3 with a sample of teachers.

## Method

### Participants

Eighty participants (68% female*; M*_*age* =_ 40.65, *SD* = 11.16) were recruited using Prolific (prolific.co), which was used instead of Mturk due to the availability of teachers on the platform. Participants were all current teachers (primary 40%, secondary 34%, or tertiary 26.25%). To facilitate recruitment of teachers we recruited internationally with a majority of the sample from the UK (76%), US (4%), and Europe (12.5%). All participants reported being fluent in English. The average number of years teaching experience was 12.66 (*SD* = 8.97, range = < 1 to 34 years). Forty teachers were randomly assigned to the High-OD condition and 40 teachers were assigned to the Low-OD condition.

### Procedure and design

The same design as the previous experiments with the distributions identical to those used in Experiment 3, that is, normally distributed with means of 80 (High-OD) and 50 (Low-OD), and a SD of 5. *Δp* was again set to zero. Additional demographic variables regarding teaching were collected prior to beginning the experiment, including grade level taught and years spent teaching. Furthermore, we asked an additional background variable ‘How often do you experiment with novel teaching strategies in your classroom?’ Participants responded on a 7-point Likert-scale ranging from ‘Almost always’ to ‘Never’. In addition to the typical aggregate predictions and efficacy ratings collected at test, we asked an additional classroom intention rating where teachers were to rate ‘Based on the evidence you have observed in this study, how likely are you to use Kalavatic teaching in your classroom, if at all?’ on a 100-point scale ranging from ‘0% – would *never* use Kalavatic teaching in my classroom’ to ‘100% – would *definitely* use Kalavatic teaching in my classroom’.

## Results and discussion

### Training data

**Fig. 11:**
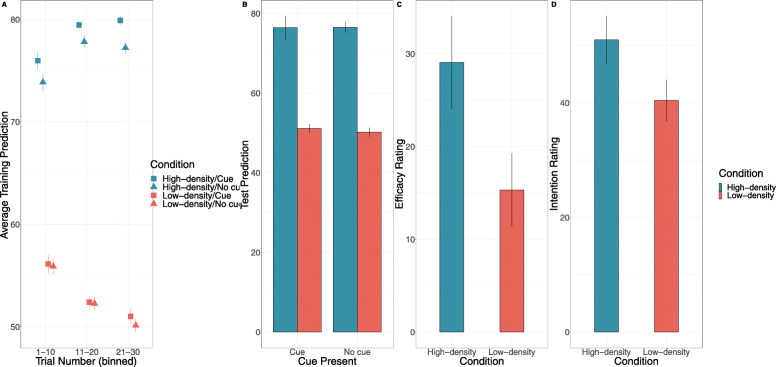
Results from Experiment 6 for **a** training predictions as a function of outcome density condition, cue presence, and trial as well as **b** predictions of students’ average performance as a function of outcome density condition and **c** efficacy ratings as a function of outcome density condition. Error bars represent + 1 standard error of the mean

Participants make significantly higher predictions when the cue was present (*M* = 65.8, *SD* = 13.43) compared to when it was absent (*M* = 64.54, *SD* = 12.64; *F* = 6.53, *p* = .013, *η*_*p*_^2^ = .01, BF_10_ = 2.67) (see Fig. 11a). Similarly, there was a significant difference in training predictions between the High-OD condition (*M* = 77.36, *SD* = 4.64) and the low outcome density condition (*M* = 52.98, *SD* = 4.27; *F* = 813.47, *p* < .001, *η*_*p*_^2^ = .91, BF_10_ > 100. The interaction between outcome density condition and the presence of the cue was marginally non-significant, *F* = 3.77, *p* = .056, *η*_*p*_^2^ = .05, BF_inc_ = 1.11.

### Aggregate predictions

As with the previous experiments, the results indicated that High-OD (*M* = 76.49, *SD* = 10.04) condition made significantly higher predictions of average performance compared to the Low-OD (*M* = 50.68, *SD* = 4.95) condition, *F* = 368.58, *p* < .001, *η*_*p*_^2^ = .83, BF_10_ > 100. There was no significant difference in predictions of average performance for Kalavatic teaching (*M* = 63.80, *SD* = 16.08) compared to ordinary teaching (*M* = 63.36, *SD* = 14.28), *F* = 0.14, *p* = .708, *η*_*p*_^2^ = .002, BF_10_ = .15, averaged across conditions. Finally, the interaction between cue type and outcome density condition was not significant, *F* = .19, *p* = .662, *η*_*p*_^2^ = .002, BF_inc_ = .15 (see Fig. 11b).

### Efficacy and classroom intention ratings

Participants in the High-OD (*M* = 29.075, *SD* = 31.69) condition made significantly greater efficacy ratings than participants in the Low-OD (*M* = 15.33, *SD* = 25.10) condition, *t* (78) = 2.15, *p* = .034, *d* = .48, BF_10_ = 1.68) (see Fig. 11c). Participants in the High-OD condition made efficacy ratings significantly higher than zero, *t* (39) =5.80, *p* < .001, *d* = .92, BF_10_ = 5.80 as did participants in the Low-OD condition, *t* (29) = 3.86, *p* = < .001, *d* = .61, BF_10_ = 3.86. This last result is contrary to Experiment 3, suggesting that while outcome density remains a significant factor in determining efficacy ratings in teachers, teachers in the low outcome condition may be a-priori more prone to making positive efficacy ratings for a novel teaching technique than participants drawn from the general population.

In addition, there was a marginally non-significant effect of outcome density on intention to use Kalavatic teaching in the classroom, *t* (78) = 1.9, *p* = .059, *d* = .42, BF_10_ = 1.12, with the High-OD condition (*M* = 51.0, *SD* = 26.33) more likely to indicate they would use Kalavatic teaching compared to the Low-OD condition (*M* = 40.43, *SD* = 22.99) (see Fig. 11d). There was a significant positive correlation between efficacy ratings and intention to use Kalavatic teaching in the classroom, *r* = .69, *p* < .001.

### Exploratory analyses

We also performed a number of exploratory analyses. First, we examined whether teaching experience moderated the outcome density effect by examining the interaction between teaching experience and condition, using each of the ratings from the test phase. Similarly, we performed the same models using the frequency with which teachers indicated that they experiment with novel teaching strategies in their classroom as a moderator. The results are presented in Tables 2 and 3. Finally, we also examined whether the outcome density effect differed as a function of teacher-grade level (primary vs. secondary vs. tertiary), using tertiary teachers as the reference category. None of the exploratory analyses suggested that the outcome density effect was significantly moderated.

**Table 2:** Results of the exploratory regressions performed using teacher background variables from Experiment 6 with efficacy rating as the criterion variable

Predictors	Beta	CI	***p***	Beta	CI	***p***	Beta	CI	***p***
Intercept			**< .001**			.855			.078
Low-OD vs. High-OD	− .34	− .72–.04	.085	.01	− .99–1.00	.99	− .12	− .54–.29	.562
Years teaching	− .16	− .44–.12	.267						
OD X years teaching	.12	− .29–.53	.554						
Novel				.25	− .06–.57	.122			
OD X novel				–.25	− 1.27–.76	.628			
Primary vs. tertiary							.42	.05–.79	**.03**
Secondary vs. tertiary.							.13	− .25–.51	.496
OD X primary vs. Tertiary.							− .2	− .62–.22	.349
OD X secondary vs. tertiary.							0	− .42–.42	.992
*R*^*2*^/*R*^*2*^ adjusted	.072/.035			.096/.061			.131/.072		

*OD* outcome density, CI = 95% confidence intervals

**Table 3:** Results of the exploratory regressions performed using teacher background variables from Experiment 6 with intention rating as the criterion variable

Predictors	Beta	CI	***p***	Beta	CI	***p***	Beta	CI	***p***
Intercept			**< .001**			.225			**< .001**
Low-OD vs. High-OD	− .33	− .72–.05	.091	− .15	− 1.15–.84	.76	− .02	− .44–.41	.942
Years teaching	0	− .28–.28	.99						
OD X years teaching	.17	− .25–.58	.429						
Novel				.25	− .06–.56	.122			
OD X novel				− .06	− 1.07–.96	.914			
Primary vs. tertiary							.39	.01–.77	**.047**
Secondary vs. tertiary							.22	− .16–.61	.258
OD X primary vs. tertiary							− .24	− .66–.19	.277
OD X secondary vs. tertiary							− .15	− .58–.28	.503
*R*^*2*^/*R*^*2*^ adjusted	.058/.021			.101/.066			.098/.037		

*OD* outcome density, CI = 95% confidence intervals

## General discussion

In a series of six experiments we examined the outcome density effect in relation to causal illusions about instructional practices. We hypothesised that the frequency of positive outcomes plays a role in promoting inaccurate beliefs about the efficacy of ineffectual instructional practices. Across all six experiments we showed that outcome density affects participants’ causal ratings such that when simulated student performance was high, participant observers were more likely to believe that a novel teaching technique was effective, despite the fact that it was either ineffective or detrimental to performance. Using a typical contingency learning paradigm, in Experiment 1 we showed that outcome density affects causal beliefs about instructional practices when the outcome is categorical. Like the vast majority of outcome density experiments, we showed that when the salient category (i.e. high performance) was more frequent, causal illusions were more likely. In Experiments 2 and 3 we examined the outcome density effect in a more educationally plausible scenario – when the outcome (i.e. students’ performance) was continuous. Both experiments indicated that when the distribution of students’ performance favoured frequent positive outcomes, participants were more likely to infer that a novel teaching technique was effective at promoting students’ academic performance. In Experiment 4 we showed that even when we controlled the contingency between the use of a novel teaching technique and students’ performance to be negative, participants who observed frequent positive outcomes continued to rate the teaching technique as effective. In Experiment 5, we demonstrated that outcome density effects similarly occur when student outcomes were presented concurrently, and, finally, in Experiment 6 we replicated the outcome density effect in a sample of teachers.

Inaccurate beliefs about the effectiveness of instructional techniques are widespread among educators (Dekker et al., 2012). Little is known, however, about the psychological mechanisms that develop and maintain these beliefs. Here, we have examined the role that contingency learning may play in maintaining false beliefs in teachers. While humans are generally good at tracking contingencies between events and outcomes (Shanks & Dickinson, 1988; Wasserman, 1990), there is clear evidence that there are robust biases that undermine these inferences (e.g. Don & Livesey, 2017; Hannah & Beneteau, 2009; Matute, Blanco, & Díaz-Lago, 2019). The current study replicates the outcome density effect in a new domain and suggests that outcome frequency may be an important factor in determining teachers’ instructional beliefs. The fact that when positive outcomes are frequent, participants’ causal inferences can vary markedly from reality, e.g. believing that an instructional practice is improving students’ performance when it is, in fact, impairing performance, has substantial implications for how teachers use classroom assessments and evaluate their students’ outcomes.

Importantly, the distributions used in the high outcome density conditions, where causal illusions tended to occur, closely mirror the distributions of typical classroom assessments used by practicing teachers. The quality of teachers’ judgements and decision-making about the efficacy of their practice will be determined by the extent to which the learning environment can provide access to informative feedback. This is particularly important because very often teachers are encouraged to frequently use classroom assessments to evaluate and improve their practice (Black & Wiliam, 1998). Indeed, assessments have been proposed to create ‘a moment of contingency’ (p. 285) where teachers are able to use the assessment as evidence and adapt their instruction accordingly (Wiliam, 2006). These propositions assume that teachers are able to accurately infer the contingency between their practice and their students’ performance on the assessment. However, if teacher’s inferences are biased by the frequency of an outcome then classroom-based assessments may have less utility as a tool for teachers to evaluate and improve their practice than proposed. Importantly, the results of these experiments make a clear, if not counter-intuitive, prediction that, all else being equal, teachers with high-performing students are going to be more susceptible to causal illusions and more likely to endorse ineffectual instructional practices. Further research is needed to examine this prediction in real-world educational contexts.

It is also worth noting that within educational research, teacher reports of efficacy are still often used as an indicator of an intervention’s efficacy, and sometimes the sole indicator of the intervention’s efficacy (e.g. Beauchemin, Hutchins, & Patterson, 2008; Berg, Bergendahl, Lundberg, & Tibell, 2003; Lage, Platt, & Treglia, 2000; Martens, Peterson, Witt, & Cirone, 1986). The current results suggest that it is difficult for researchers to rely on such reports to evaluate efficacy as they are influenced by the base rate of student performance (along with other biases such as demand characteristics). It has been suggested that there is a weak link between practice in schools and teacher education world-wide. Teacher students are provided with theory by their teacher educators which is not sufficiently integrated into classroom experiences, leading to the long-standing mismatch between theory and practice (Lillejord & Børte, 2016; Zeichner, 2010).

Detecting causal relationships within the classroom is a difficult task. Teachers are faced with discerning the relationship between a large number of outcomes, across both students and time, with a multitude of putative causes. This is in addition to various other complexities such as moderation, mediation, and autocorrelation. Within such a complex environment, individuals are likely to perceive meaningful patterns where there is only random noise (Gilovich, 1991). While, in some situations, this tendency may be less costly to the individual than failing to detect a pattern in the environment, it might also produce behaviours that are simply redundant (Blanco, 2017). In addition, as shown in Experiment 4 it may cause participants to fail to detect that a new practice is actually worsening performance, which may come at a considerable cost to students’ achievement. It should also be acknowledged that most previous research on how teachers learn, has focussed upon how practice can be better linked to theory, and not the other way around (Korthagen, 2017).

One explanation for this bias in contingency learning is that the tracking of cue-outcome contingencies is resource-intensive and requires the learner to attend to, and calculate on-line, the independent relationships between the cue and the outcome, and the outcome without the cue present (De Houwer, 2009; Mitchell, De Houwer, & Lovibond, 2009). To overcome this high cognitive demand, people rely on heuristics to estimate contingencies between events which can result in overestimating the frequency of salient events (Fiedler et al., 2009). In this case, evidence suggests that people often pay substantially more attention to instances where the cue and expected outcome co-occur (i.e. confirmatory trials; Crocker, 1982; Jenkins & Ward, 1965), which may, in turn, create inaccurate intuitions when the frequency of such confirmatory trials is high. This bias to attend to confirmatory trials appears to also be present when trial information is summarised, as in Experiment 5. Although one may argue that the presentation format is still sequential in nature—participants only ever saw one cue type on each screen—the cognitive resources required to track and compute average student performance for Kalavatic teaching and ordinary teaching online is greatly reduced in this format. Nevertheless we still find participants showing the tendency to overestimate the efficacy of Kalavatic teaching when the base rate of student performance is high than when it is low, even when student performance was equivalent under ordinary teaching methods.

In all six experiments, the clear outcome density effects observed in ratings of the efficacy of the novel educational practice were not reflected in cue-specific predictions made during training, nor judgements of mean outcome in the presence and absence of the novel training made during testing. In some instances, participants gave higher predictions in the presence of the cue than in its absence (consistent with an illusory causation effect) but the strength of this difference was equivalent in Low-OD and High-OD conditions. This is not an uncommon finding in research on the outcome density effect, which is typically observed more clearly on causal and efficacy ratings than on other measures. In other instances, particularly when the outcome presented is continuous and there is no meaningful contingency between cue and outcome, there was little evidence of any illusory causation in these predictions. It is worth noting that the aggregate predictions closely resemble the ratings during training, to which participants receive extensive feedback. The lack of outcome density effect on these ratings may reflect that through the training process participants have learnt to accurately make predictions about the expected outcome. Indeed, it is interesting that despite often making these aggregate predictions accurately, participants then make inaccurate efficacy ratings.

Some researchers have argued that this disassociation between prediction ratings and causal ratings indicates the involvement of a different system or psychological process when participants are making predictions rather than causal judgements (Allan, Siegel, & Tangen, 2005; Blanco & Matute, 2015; Waldmann, 2001). However, given that this is a single dissociation where the effect is reliable on one measure and inconsistent on the other, it may simply come down to the sensitivity of each individual measure to subtle biases (De Houwer, Vandorpe, & Beckers, 2007; Vadillo et al., 2005; Vadillo, Musca, Blanco, & Matute, 2011). Ultimately, the extent to which aggregate predictions or causal ratings predict actual classroom behaviour is an empirical question requiring further investigation.

In any case, it is important to note that the type of measure on which outcome density effects have most reliably been observed in other contexts also generated outcome density effects in this educational context. An important assumption in causal learning research is that causal and efficacy ratings reflect beliefs about treatments, interventions, or practices that have real consequences for people’s choices. That is, we choose whether or not to purchase a treatment or engage in an activity because we believe that doing so will cause a desirable outcome. It remains to be seen whether illusory causation in this context translates to real-world implications for teachers’ decisions to adopt new educational practice. With this in mind, it is noteworthy just how influential some educational practices have been despite their poor evidence base and apparent lack of any causal effectiveness in the classroom (Bruyckere et al., 2015; Kirschner et al., 2006).

The conditions that give rise to the outcome density effect are likely to be encountered in everyday life and, indeed, may be desirable in some instances. For instance, the outcome density effect occurs in medical learning scenarios when patients frequently spontaneously recover. For this reason, researchers have highlighted the relevance of the effect to the popularity of complementary and alternative medical treatments for mild ailments that are likely to spontaneously remit without treatment (e.g. *Echinacea* use for the common cold). This has led some to comment that we can do nothing to change the outcome density effect (Matute et al., 2015). In education, we obviously want students to perform as well as possible, presenting the same challenge in reducing outcome density effects. However, these findings suggest that if a teacher has a large number of high-performing students, they may have difficulty identifying optimal teaching methods that will help those not performing well.

Some general solutions to biases in contingency learning have been proposed. For instance, Vadillo, Matute, and Blanco (2013) showed that if there are reliable alternative causes for an outcome then individuals are less prone to the outcome density effect. It might, therefore, be worthwhile to stress to teachers that when considering their students’ performance on assessments they need to bear in mind factors other than their teaching that will affect the outcomes (e.g. natural ability, maturation, the difficulty of the assessment, etc.). Another potential avenue for correcting biased causal judgements is to provide explicit base rate expectancies regarding student performance, particularly when the student cohort is high achieving and, therefore, academic performance is negatively skewed (i.e. most students will perform well). In a study by Blanco and Matute (2019, Experiment 1), participants were either pre-trained to expect a high outcome base rate or not pre-trained in a zero-contingency learning task; they found that participants who were exposed to a high base rate in pre-training showed reduced illusory causation in causal judgements compared to control participants, despite witnessing an identical high base rate zero-contingency cue-outcome relationship. In a subsequent study, they showed the reverse effect, where participants who were pre-trained on a low outcome base rate showed an inflated illusory causation effect relative to control participants (Blanco & Matute, 2019, Experiment 2). These findings suggest that causal illusions can be influenced by prior expectancies about the base rate of outcome occurrence. Although not measured in the current set of experiments, it would be interesting to see if teachers’ prior expectations about student performance influenced their susceptibility to cognitive biases such as the outcome density effect. However, it is also worth mentioning that teachers’ misconceptions are often resistant to interventions designed to correct them. In one study, Ferrero, Hardwicke, Konstantinidis, and Vadillo (in press) provided educators with texts to refute their misconceptions. While the intervention affected short-term beliefs, it had no effect in the long-term (after 30 days) and disconcertingly *increased* the extent to which teachers indicated that they were willing to implement educational practices based on the misinformation.

Outcome density effects as they relate to educational assessment may offer additional opportunities for intervention. For example, a relatively simple solution in practical terms might be to set more difficult classroom assessments, thereby artificially reducing the frequency of high-achieving students. However, such an intervention would need to be evaluated against the risk of potentially negative side effects (e.g. negative impacts on student self-efficacy). Another potential focus for intervention could be on how such results are communicated to teachers and potentially utilising ranks or standardised scores rather than grades or percentage-correct-type feedback. While the present study cannot speak to the efficacy of such interventions, these appear to be plausible and worthwhile avenues for further research.

While the current study has shown the outcome density affects causal ratings about educational practices in an experimental paradigm, further research is needed to examine how well such findings generalise to the classroom. In particular, the current study did not utilise real teachers as participants. While we have no reason to suspect that teachers are likely to be particularly susceptible or immune to the contingency learning mechanisms responsible for this bias, teachers may have particularly strong prior beliefs and expectations that may affect how causal illusions play out in the classroom (Mutter, Strain, & Plumlee, 2007; Yarritu & Matute, 2015). Furthermore, teachers have access to not only trial-by-trial observation in the case of educational assessments, they have additional information such as means and distributions. This information may allow teachers to make more accurate inferences, particularly if teachers receive training in the scientific method. However, in some circumstances it may make the effect worse because teachers may confound aggregate level correlations with student-level ones (Fiedler et al., 2007). The findings of Experiment 5 suggest that presenting outcomes concurrently as might often occur as a teacher monitors a class’ performance, did not change the underlying pattern of results such that high outcomes promoted causal illusions. While there are many conceivable ways that assessment information can be aggregated in an educational context, these findings suggest that receiving information about multiple outcomes does not *necessarily* eliminate the outcome density effect.

The current study has shown that the outcome density effect can be applied to causal inferences about the efficacy of teachers’ instructional practices. In six experiments we have shown that, when students frequently perform well, participants tend to infer that a novel teaching method is working despite the fact that it either has no effect or a negative effect. This suggests that teachers may have difficulty using classroom assessments to evaluate the efficacy of their practice. This is especially true because most classroom assessments are designed so that a majority of students perform well, providing a context where the outcome density effect is particularly likely. While further research is needed to examine how such phenomena play out in the classroom, the current study provides a plausible mechanism for understanding why teachers often believe in the efficacy of practices that have little scientific support.

## Data Availability

The datasets generated and/or analysed during the current study are available in the Open Science Framework repository, https://osf.io/afcr2/.
